# Herbivory and Stoichiometric Feedbacks to Primary Production

**DOI:** 10.1371/journal.pone.0129775

**Published:** 2015-06-22

**Authors:** Jennifer Adams Krumins, Valdis Krumins, Eric Forgoston, Lora Billings, Wim H. van der Putten

**Affiliations:** 1 Department of Biology and Molecular Biology, Montclair State University, Montclair, New Jersey, United States of America; 2 Department of Environmental Sciences, Rutgers University, New Brunswick, New Jersey, United States of America; 3 Department of Mathematical Sciences, Montclair State University, Montclair, New Jersey, United States of America; 4 Department of Terrestrial Ecology, Netherlands Institute of Ecology, Wageningen, The Netherlands; 5 Laboratory of Nematology, Wageningen University, Wageningen, The Netherlands; Dowling College, UNITED STATES

## Abstract

Established theory addresses the idea that herbivory can have positive feedbacks on nutrient flow to plants. Positive feedbacks likely emerge from a greater availability of organic carbon that primes the soil by supporting nutrient turnover through consumer and especially microbially-mediated metabolism in the detrital pool. We developed an entirely novel stoichiometric model that demonstrates the mechanism of a positive feedback. In particular, we show that sloppy or partial feeding by herbivores increases detrital carbon and nitrogen allowing for greater nitrogen mineralization and nutritive feedback to plants. The model consists of differential equations coupling flows among pools of: plants, herbivores, detrital carbon and nitrogen, and inorganic nitrogen. We test the effects of different levels of herbivore grazing completion and of the stoichiometric quality (carbon to nitrogen ratio, C:N) of the host plant. Our model analyses show that partial feeding and plant C:N interact because when herbivores are sloppy and plant biomass is diverted to the detrital pool, more mineral nitrogen is available to plants because of the stoichiometric difference between the organisms in the detrital pool and the herbivore. This model helps to identify how herbivory may feedback positively on primary production, and it mechanistically connects direct and indirect feedbacks from soil to plant production.

## Introduction

When herbivores consume plants, either above or below ground, they release mineralized nutrients into the environment supporting microbial activity and enhancing primary production [1–3]. This occurs through three potentially concurrent mechanisms. First, plants may compensate for herbivory with additional growth [4]. Second, due to a stoichiometric imbalance between the herbivore and the plant, a herbivore may excrete excess mineral nutrients into the surrounding environment. Generally, when consumers eat a resource with a lower carbon to nitrogen (C:N) ratio they contribute excess mineral nutrients (N) to the soil (*e*.*g*. protists or nematodes eating bacteria [5]). However, when they consume something relatively depleted in N (a higher C:N), they contribute excess organic carbon to the soil [6–9]. Third, herbivores do not always completely ingest vegetation. As they graze, leaf material above ground or root exudates and particles below ground may not be consumed fully. The ‘partial feeding’ of the herbivore results in discarded and non-ingested detrital plant material that potentially primes the soil by increasing the quantity of organic matter and therefore, feeding microbial metabolism within the detrital pool [10–13]. These mechanisms are not mutually exclusive; in particular, the third is dependent on the principles of the second. Whether or not the excess organic matter is ingested, decomposed and mineralized by the herbivore or in detritus depends on the fraction of biomass consumed by the herbivore. Following that, the stoichiometric imbalance between plant material and the detrital microbes or herbivores will determine whether organic carbon accumulates or mineral nitrogen accumulates [14]. These facts determine whether or not the plant will benefit from the indirect soil-priming effects [12] of a herbivore that consumes only a fraction of what it grazes or suffer from the direct consumptive effects of a herbivore that consumes the plant whole.

**Fig 1:**
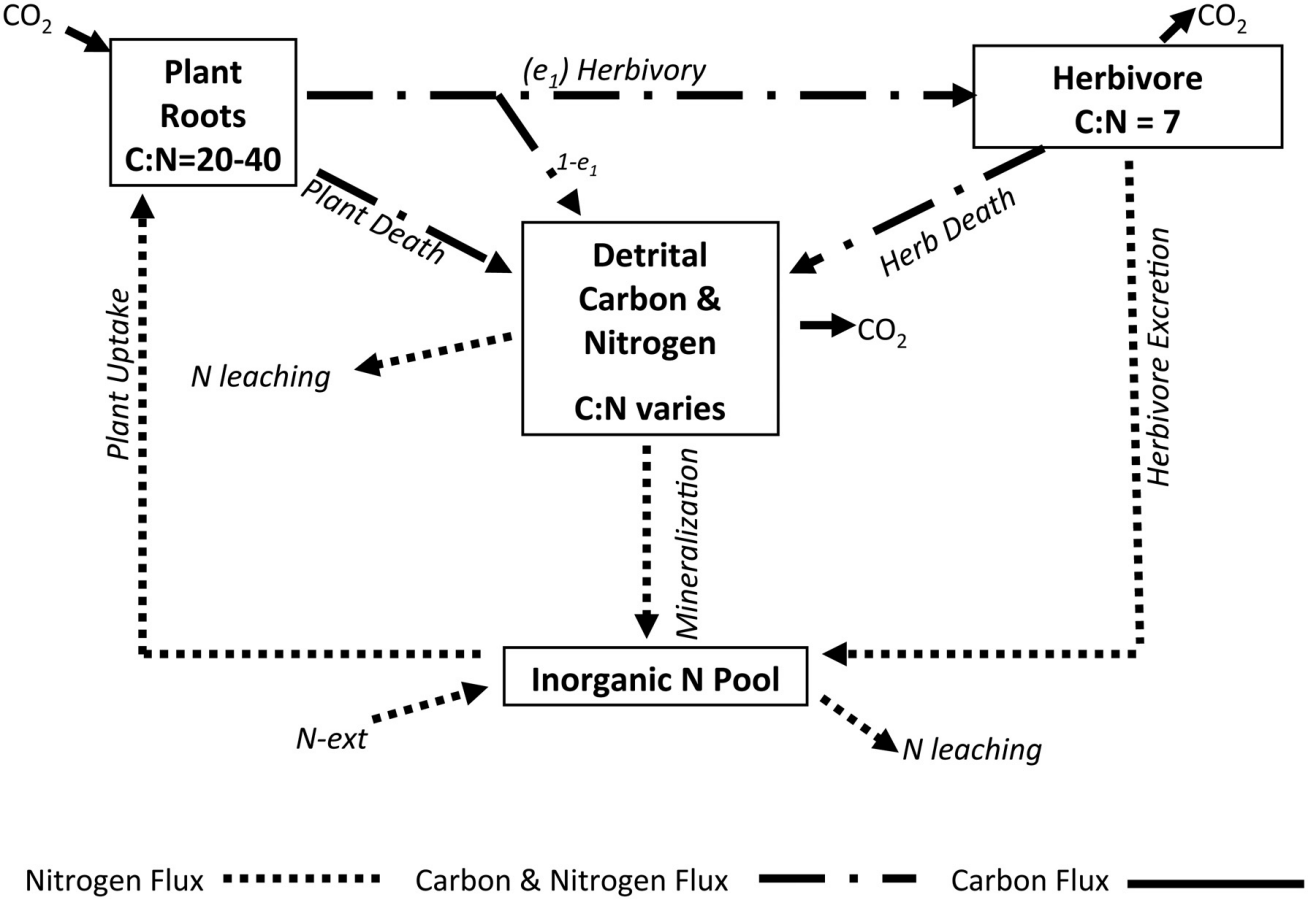
Diagram of pools and fluxes for our model indicating the flow of carbon only (solid lines), nitrogen only (dotted lines) and carbon and nitrogen both (dotted and dashed lines).

Organic material released during herbivory imposes a direct cost to the plant. However, herbivores can be highly selective or even sloppy, consuming only a fraction of what is grazed and sometimes only the highest quality fraction [15]. In this case, partial feeding will function in an ecosystem differently than efficiency in the sense described by Lindeman [16], where only some fraction of energy and nutrients consumed on one trophic level are transferred to the next higher trophic level. Rather, the fraction of un-consumed organic matter left behind in the form of plant fragments or root exudates and particles is not assimilated by the herbivore. However, it is not completely lost to the plant because even though the un-consumed plant biomass does not support herbivore biomass, it will indirectly benefit the plant when it is decomposed and mineralized in the detrital pool (Fig 1).

Early extensive work set the foundation for herbivory theory in aboveground communities [17] that has since been extended to the rhizosphere, where nutrient cycling takes place [1]. Past work has shown that roots can undergo compensatory growth in the same way as above ground biomass [18]. However, this may not be a purely plant physiological response. Instead, the mechanism behind this effect is likely explained by metabolic processes mediated by microbes in the detrital pool of the soil. In one experiment, when nematodes grazed roots, organic nutrients were released and microbial nitrogen cycling increased [19]. The stoichiometric quality of the organic matter as it is digested by the herbivore or decomposed and mineralized by microbial consumers will determine the benefit to the plant [6], and mechanisms of herbivory vary. Some herbivores can consume biomass whole when they graze while others leave large amounts of plant biomass behind as detritus. Our model addresses this variation and accounts for multiple pathways of decomposition such that it helps to realistically describe the mechanism.

Our model explores the idea that “partial feeding”, defined as *e*_*1*_, of a herbivore interacts with the stoichiometric quality of the grazed plant to determine metabolism in the detrital pool and nutritive feedback to plants. Specifically, the model enables the study of the relative effects of the quantity of organic matter priming the soil versus the stoichiometric imbalance between the consumer and the consumed (relative quality of organic matter) [10]. This interaction is important, and can be extended when one considers foraging activity and consumptive patterns of different types of herbivores. For instance, herbivorous nematodes in soil may draw root material through a spear or stylet leaving only dissolved organics behind if at all. In contrast, chewing insects are likely to leave large particulate matter behind. Our model was inspired by soil food web models (*e*.*g*. van Veen, et al. [20]), and our parameter values were taken from previous soil food web models and our own green house studies (Table 1). However, with appropriate parameter values, the theory and application presented here may easily be extended to other plant—herbivore interactions.

**Table 1:** Variable and parameter definitions, values and their references.

**Parameter**		**Value**	**Units**	**Notes**
*CNP*	C:N ratio of plants	varies 20–40	g C · g N^-1^	[8]
*CNH*	C:N ratio of herbivores	7	g C · g N^-1^	[9]
*e_1_*	Herbivore Partial Feeding	varies 0.1–1.0	—	The fraction of plant material digested by the herbivore vs. lost to the detrital pool.
*r_P_*	Specific plant growth rate (funct. of N)	281	g soil · g N^-1^ d^-1^	= 0.0722/ N [27]
*d_P_*	Plant death rate	0.003	d^-1^	[33]
*r_1H_*	Specific herbivory (function of P and H)	100	g soil · g C^-1^ d^-1^	[33]
*d_H_*	Herbivore death rate	0.003	d^-1^	[34]
*r_2H_*	Herbivore respiration rate	0.014	d^-1^	Estimate based on approximate 1% biomass carbon respired
*r_min_*	mineralization rate	5.8 · 10^-3^	d^-1^	estimate based on labile carbon [44]
*k_NL_*	N leaching coefficient (labile N pool)	0.0095	d^-1^	based on greenhouse experiment: assumes 1L soil, watered 100 mL 2x/week; 10% of applied water goes out bottom.
*k_DL_*	N leaching coefficient (detrital pool)	0.001	d^-1^	= 0.1 * kNL
*N_ext_*	External N addition	2.7·10^-6^	g N · g soil^-1^ d^-1^	Standard 0.1X Hoagland Solution used in greenhouse experiment [25]
	**State Variable**		**Units**	
*P*	Plant Biomass		g C · g Soil ^-1^	
*H*	Herbivore Biomass		g C · g Soil ^-1^	
*N*	Mineral Nitrogen		g N · g Soil ^-1^	
*D_C_*	Detrital Carbon		g C · g Soil ^-1^	
*D_N_*	Detrital Nitrogen		g N · g Soil ^-1^	

## Materials and Methods

### Model Development and Assumptions

The model simulates a system with seven interacting pools: plants (*P*_*C*_ and *P*_*N*_ referring to plant carbon biomass and plant nitrogen biomass respectively), herbivores (*H*_*C*_ and *H*_*N*_ referring to herbivore carbon biomass and herbivore nitrogen biomass respectively), detritus (*D*_*C*_ and *D*_*N*_ referring to detrital carbon and nitrogen respectively), and inorganic nitrogen (*N*). However, the stoichiometry of plants and herbivores is fixed such that *P*_*N*_ = *P*_*C*_ */ CNP* and *H*_*N*_ = *H*_*C*_ */ CNH*, so only five pools can independently vary (*P*_*C*_, *H*_*C*_, *D*_*C*_, *D*_*N*_ and *N*) (Fig 1). Our model was developed using the following simplifying assumptions:

1. The plant pool includes both shoots and roots as a single unit. It net assimilates CO_2_, and an important assumption of this model is nitrogen limitation [21]. Therefore, carbon assimilation is limited by the availability of nitrogen, and following Liebig’s Law [22], an organism will only assimilate nutrients in proportion to that nutrient which is most limiting. Therefore, nitrogen limitation affects the degree to which a plant can photosynthesize and grow [23]. Further, because the system is assumed to be nitrogen limited, the model does not account for the plants threshold elemental ratios (TER).
2. The C:N values of the herbivores are fixed.
3. Excretion by the herbivore is explicitly modeled here, but egestion is not as it has been done in other models [6]. To maintain simplicity, we assume egestion (any undigested waste) of the herbivore will retain the C:N value of its food (*CNP)* and enter the detrital pool as unconsumed plant biomass does following *1-e*_*1*_.
4. We have incorporated the microbial (bacterial and fungal) pools and their functioning into the detrital pool. This is an important simplification that assumes the detrital pool encompasses a microbial consortium that includes fungi and bacteria and is capable of decomposition and mineralization [24]. However, this model can be used as a framework on which additional processes may be resolved such as the capacity of the microbial pool to either assimilate or mineralize nutrients, and the degree to which the detrital pool is dominated by fungal or bacterial decomposers will be reflected in the pool’s stoichiometry.

Baseline parameter values can be found in Table 1. The parameters are obtained from literature in plant and soil communities or from operating conditions used in our own greenhouse experiments. The parameter values for *R*_*2H*_ and *r*_*min*_ are estimated because they would be difficult to measure. The pools exchange carbon and nitrogen in accordance with the flux equations that account for (Fig 1): herbivore respiration, herbivore excretion and herbivore death, mineralization of detritus, herbivory, external nitrogen and plant uptake of carbon and nitrogen, and plant death. Further, nitrogen can leach from the detrital pool as organic nitrogen and from the inorganic nitrogen pool as mineral nitrogen. Organic carbon does not leach from the detrital pool, but it can be mineralized and respire out as CO_2_.

The mass balance for carbon in the plant pool is

$$\frac{{dP}_{C}}{dt}\;=\;r_{P}P_{C}N\;-\;d_{P}P_{C}-r_{1H}H_{C}P_{C},$$

where *r*_*P*_ is the relative plant growth rate which determines carbon uptake by the plant, *d*_*p*_ is the plant death rate, and *r*_1H_ is the specific herbivory rate which determines the amount of herbivory. Plant nitrogen biomass is scaled to the plant carbon biomass by the plant’s C:N ratio *CNP* so that the mass balance for nitrogen in the plant pool is

$$\frac{{dP}_{N}}{dt}\;=\;\frac{1}{CNP}(r_{P}P_{C}N\;-\;d_{P}P_{C}-r_{1H}H_{C}P_{C}).$$

The mass balance equation for herbivore carbon is

$$\frac{{dH}_{C}}{dt}\;=\;e_{1}\;(r_{1H}H_{C}P_{C})-\;d_{H}H_{C}-\;r_{2H}H_{C},$$

where *e*_*1*_, as stated, is the partial feeding of the herbivore, which determines how much plant biomass is consumed by the herbivore and how much is left behind to enter the detrital pool. The herbivore death rate is *d*_*H*_, and *r*_*2H*_ is the respiration rate of the herbivore. As in the plants, herbivore nitrogen biomass is scaled to the herbivore carbon biomass by the herbivore’s C:N ratio (*CNH)* so that the mass balance for nitrogen in the herbivore pool is

$$\frac{{dH}_{N}}{dt}\;=\;\frac{1}{CNH}(e_{1}\;(r_{1H}H_{C}P_{C})-\;d_{H}H_{C}-\;r_{2H}H_{C}).$$

The equation for herbivore nitrogen can be algebraically rearranged to account for herbivore excretion. Excretion does not include the nitrogen flux from the plants to the herbivores or the nitrogen flux out of the herbivore pool due to death. Therefore, we quantify herbivore excretion by

$$(\frac{1}{CNP}-\frac{1}{CNH})e_{1}r_{1H}H_{C}P_{C}\;+\frac{1}{CNH}r_{2H}H_{C}.$$

The equation for nitrogen fluxes into or out of the inorganic nitrogen pool is given by

$$\frac{dN}{dt}\;=\;N_{ext\;}+r_{min}D_{N}+\;(\frac{1}{CNP}-\frac{1}{CNH})e_{1}r_{1H}H_{C}P_{C}\;+\frac{r_{2H}H_{C}}{CNH}-\frac{r_{P}P_{C}N}{CNP}-K_{NL}N,$$

where *N*_*ext*_ is an external input of nitrogen (*e*.*g*. Hoagland solution [25]), *r*_*min*_ is a first-order mineralization rate constant that determines how much nitrogen is released from the detrital nitrogen pool, and *K*_*NL*_ is a first-order nitrogen leaching constant. Plant uptake of nitrogen is reflected in the subtraction of the term *r*_*P*_ *P*_*C*_ *N* with respect to *CNP*.

Following Fig 1 and using terms from the preceding equations, the detrital carbon and nitrogen pools are modeled as

$$\frac{{dD}_{C}}{dt}\;=\;d_{P}P_{C}\;+\;d_{H}H_{C}\;+(1-e_{1})r_{1H}H_{C}P_{C}–r_{min}D_{C},$$

and

$$\frac{{dD}_{N}}{dt}\;=\;\frac{d_{P}P_{C}}{CNP}+\;\frac{d_{H}H_{C}}{CNH}+\frac{1}{CNP}(1-e_{1})r_{1H}H_{C}P_{C}-\;r_{min}D_{N}-\;K_{DL}D_{N},$$

where *K*_*DL*_ is a first-order detrital nitrogen leaching constant.

All of our equations follow a type I functional response. This was established primarily to maintain simplicity of a complex model. However, because the model operates under the assumption of resource limitation, it is appropriate and follows other models with complementary goals [6,26].

We analyze the system of differential equations described above by solving for the steady states. We find three solutions describing the following settings: 1. all living pools are extinct, 2. herbivore extinction, and 3. the co-existence solution in which all pools exist (see equations in S1 Text). In the remainder of the paper, we are interested in exploring the third steady state solution in which all pools coexist. To determine the stability of the solution, we first computed the Jacobian matrix (S2 Text) of the mass balance equations. Substitution of the steady state solution into the Jacobian leads to extremely complicated expressions for the eigenvalues, making an analytical study of the stability intractable. Therefore we numerically compute the eigenvalues and determine that the coexistence steady state solution is stable for our baseline parameter values and for wide ranges of input parameter values tested (20 ≤ *CNP* ≤ 40) and (0.1 ≤ *e*_*1*_ ≤1).

### Model Sensitivity

We focus the analysis in this manuscript on the sensitivity of herbivore *e*_*1*_ and the interaction of *e*_*1*_ and stoichiometric quality of the host plant, *CNP*. In so doing, we are exploring the idea that more complete herbivore consumption will lead to decreased primary production by decreasing soil organic matter availability and therefore mineral nitrogen. We explore this idea by varying the efficiency with which the herbivore pool grazes at three different levels of plant quality defined by: *CNP* = 20, 30 and 40. We choose these C:N values because they bound a reasonable range of quality for grasses (we used *Ammophila arenaria* to parameterize the model [27]). By definition, 0 ≤ *e*_*1*_ ≤1.

The model is sensitive to the values of *r*_*2H*_, *CNP* and *CNH* (see S3 Text for an analytical explanation) because excretion, as defined in Fig 1, must be greater than or equal to zero.

## Results and Discussion

**Fig 2:**
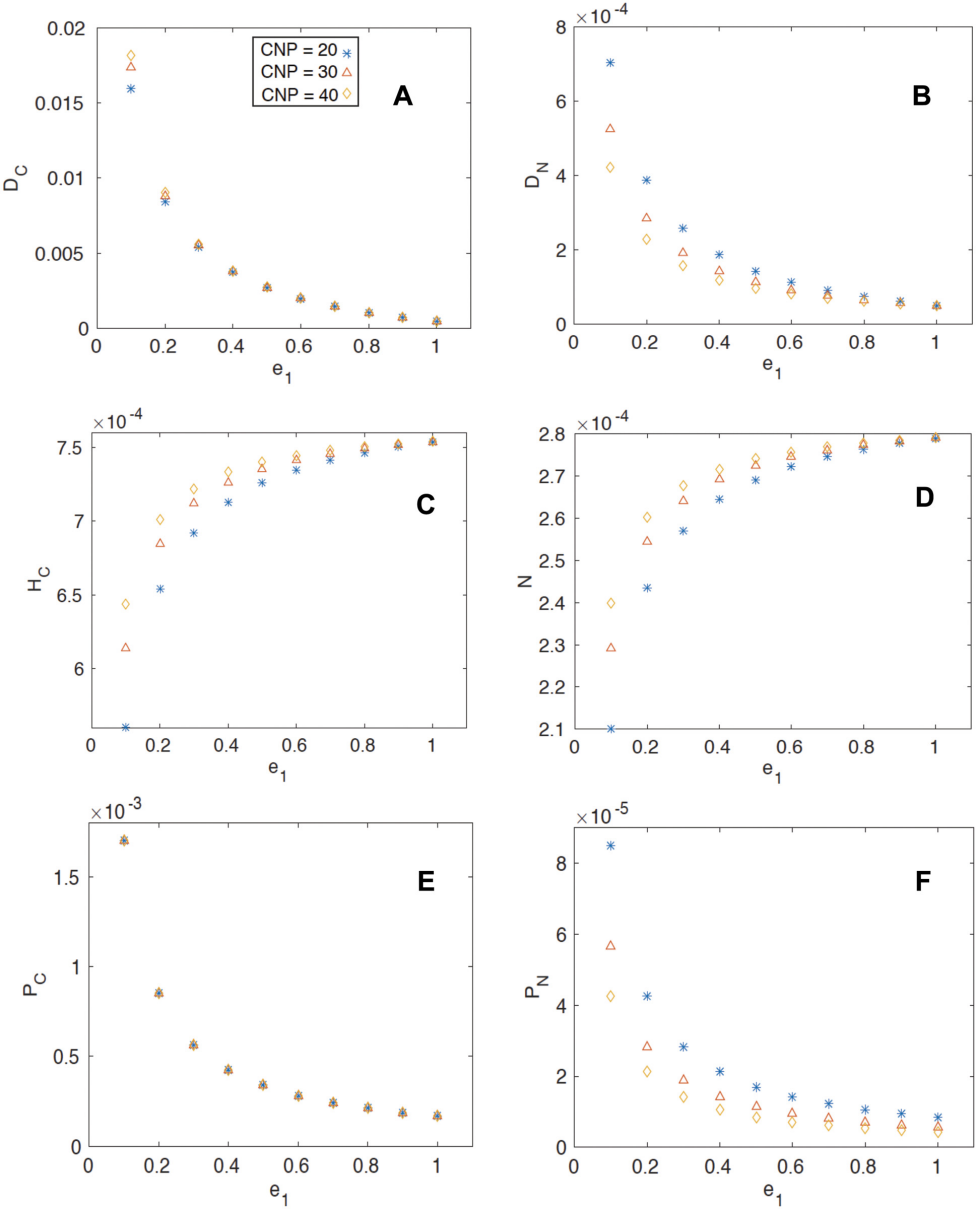
The steady state coexistence solution of the pools as a function of herbivore efficiency, a. detrital carbon, b. detrital nitrogen, c. herbivore biomass carbon, d. inorganic nitrogen and e. plant biomass carbon and f. plant biomass nitrogen. In all panels, asterisks indicate *CNP* = 20, triangles indicate *CNP* = 30 and diamonds indicate *CNP* = 40.

**Fig 3:**
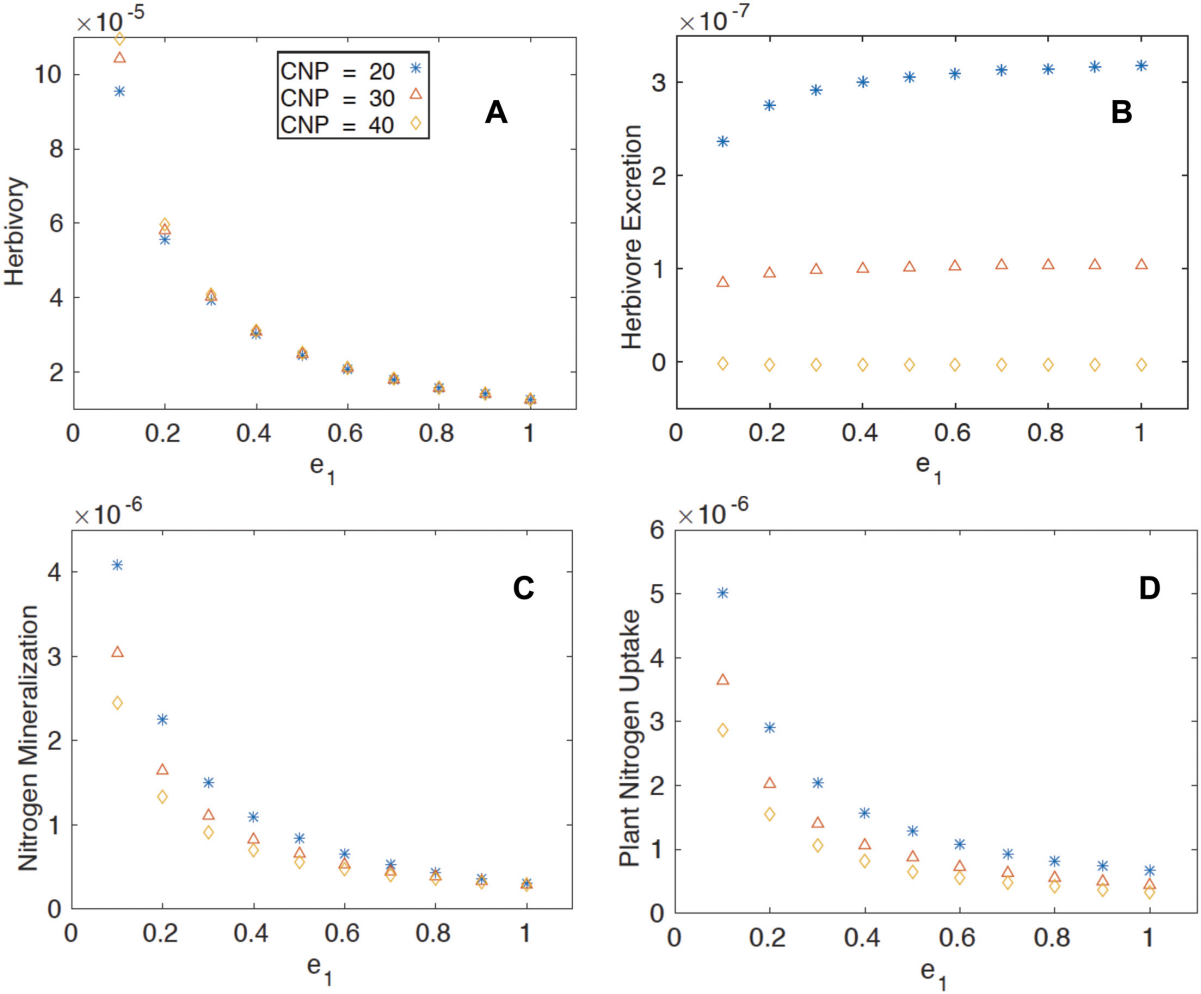
The steady state coexistence solution of the fluxes as a function of herbivore efficiency, a. herbivory, b. herbivore excretion, c. nitrogen mineralization and d. plant uptake. In all panels, symbols are presented as in Fig 2.

The goal of this work was to resolve the mechanisms through which herbivory can be beneficial to plant growth. We explore the idea that the benefits of herbivory would vary depending whether nutrients are mineralized through the herbivore or the detrital pools with respect to the stoichiometric quality of the plant grazed. The results show that when *e*_*1*_ is low, the amount of detrital carbon and nitrogen present in soil increases, and therefore the proportion of plant biomass mineralized in the detrital pool as opposed to the herbivore pool increases. The availability of detrital carbon (Fig 2a) is highest when *e*_*1*_ is very low and the C:N of the plant is high, and the difference between available detrital carbon from plants of low C:N and those from high C:N declines with increasing *e*_*1*_. Intuitively, the reverse is true for detrital nitrogen (Fig 2b) in that more is available when plant C:N is lowest, but again, this interacts with *e*_*1*_. We see that when *e*_*1*_ is greatest, herbivore biomass is greatest (Fig 2c), and that this interacts with plant stoichiometry in that herbivore biomass is greatest when they consume high C:N plants, but this difference decreases as *e*_*1*_ increases. The availability of inorganic nitrogen for plant uptake increases as herbivore *e*_*1*_ increases (Fig 2d). It follows that inorganic nitrogen availability is highest at more complete herbivore feeding (high *e*_*1*_) because plant biomass (Fig 2e and 2f), and therefore plant uptake (Fig 3d), are lower. Plant biomass nitrogen is lower because herbivore biomass is higher (Fig 2c); This difference is most pronounced when plant C:N is lowest (Fig 2f).

With respect to the fluxes, herbivory (the loss of plant biomass due to herbivores) declines with higher *e*_*1*_ (Fig 3a because, while herbivore biomass is greater (Fig 2c), plant biomass is lower (Fig 2e and 2f). Mathematically, herbivory is determined by plant and herbivore biomass. Specifically, because *CNH* is constant, the C:N mismatch between plants and herbivores grows as the value of *CNP* rises. When one views the model output (Fig 3a), at the highest value of *CNP*, where the C:N difference will be greatest, herbivory is highest. However, this only occurs for the sloppiest grazers when *e*_*1*_ < 0.2 and the difference in herbivory as related to plant quality disappears. This suggests that herbivores that leave much biomass behind may graze more to compensate when plant quality is lower (S3 Text). Excretion by the herbivores increases as *e*_*1*_ increases and the herbivore metabolizes more plant biomass, but it is especially so at the lowest plant C:N values (Fig 3b) when there will be more nitrogen. Nitrogen mineralization occurs to the greatest extent when *e*_*1*_ is low (Fig 3c) and the detrital nitrogen pool is greatest (Fig 2b). Likewise, plant uptake of C and N is highest (Fig 3d) when plant biomass is greatest (Fig 2e and 2f). The increased plant demand helps to explain the lower levels of inorganic nitrogen at low *e*_*1*_ (Fig 2d) but high detrital nitrogen (Fig 2b). Mathematically and ecologically, this is a logical outcome. When plant biomass is greater, its capacity of plants to absorb mineral nutrients will increase.

The availability of inorganic nitrogen in soil increases with *e*_*1*_. Further, the availability is greatest when high C:N plants are consumed at low *e*_*1*_ (Fig 2d). This seems counterintuitive given that detrital nitrogen decreases as herbivores consume more biomass when they graze (Fig 2b). It can be explained two ways. First, higher *e*_*1*_ means less plant biomass to be mineralized in the detrital pool. The organic matter instead is mineralized through the metabolic proceses and excretion of the herbivore. The increase in inorganic nitrogen with higher *e*_*1*_ follows herbivore excretion that increases as the herbivore successfully ingests more material. At the same time, nitrogen mineralization in the detrital pool declines with higher *e*_*1*_ because there is less detritus available. The second explanation reflects a logical feedback between plant and soil nutrients. As plant biomass is greater (Fig 2e and 2f), the demand on soil nutrients is that much greater. The process of herbivore excretion (Fig 3b), and nitrogen mineralization (Fig 3c) are both greatest at low *CNP*. When plants capitalize on this resource, mineral nitrogen is held in the plant pool, and hence, lower levels of inorganic nitrogen are realized at lower *CNP* (Fig 2d).

Herbivory leads to mineralization of nutrients through two pathways, direct and indirect. Directly, herbivores consume organic matter and excrete mineral nutrients as waste, and indirectly, organic matter left behind when herbivore activity primes microbial mineralization of nutrients in the detrital pool. The principle driving these results is the stoichiometric mismatch between a plant and its herbivore. Indeed, the greater the degree of mismatch, the greater the intensity of herbivory will be [28] due to the need to acquire the resource in short supply. Given these challenges, plants of higher quality are more likely chosen by herbivores [29]. Herbivore consumption can directly affect plant quality [30] and indirectly affect plant quality through feedbacks from nutrient cycling [26,30]. However, whether or not the herbivore can ingest and assimilate what is consumed affects *e*_*1*_ and interacts with plant stoichiometric quality. Our model builds the connection between the direct and usually negative effects of being grazed, and the indirect benefits of mineralization emerging from the detrital pool [31]. Sloppy herbivores may graze more plant material to acquire needed nutrients; this is even more so in the case where plant quality is low. This is not inconsistent with the fact that high quality plants will be chosen more than low quality plants [29]. However, our model does not allow for herbivore choice. It does show that plant quality interacts with *e*_*1*_ to affect mineral nitrogen availability. However, the logistics of consuming low quality plant (*e*.*g*. high lignin content) material, or plants that contain defensive secondary metabolites can drive the degree to which a plant is grazed. The extent to which low quality or high quality plant material is more likely to be leaky or discarded during grazing will certainly affect the metabolism and the threshold elemental ratio of the herbivore [32]. This would be an interesting next step in the future development of this model.

**Fig 4:**
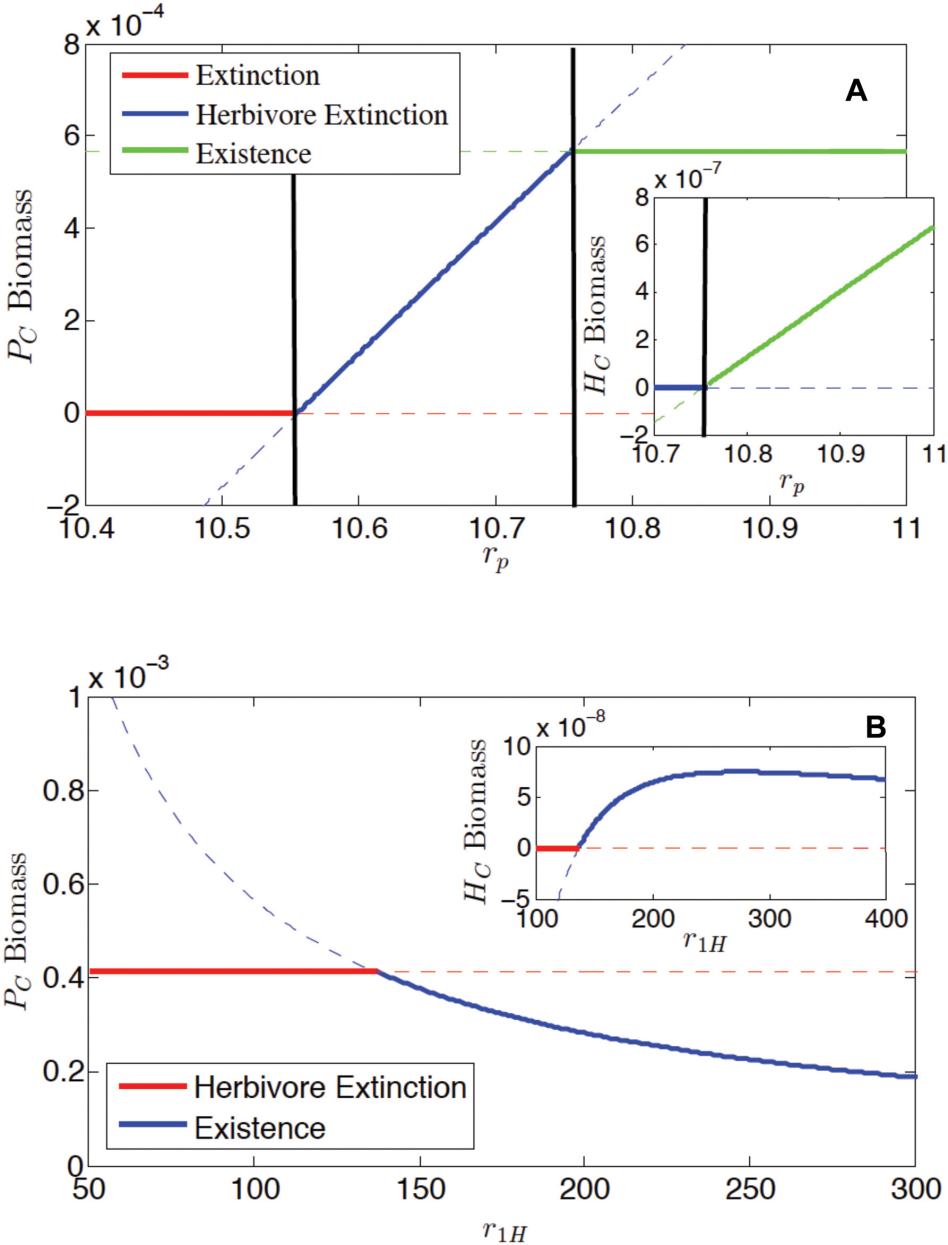
Bifurcation diagram indicating the stability and instability of the three steady state solutions as a.) *r*_*P*_ is varied, and b.) *r*_*1H*_ is varied. In both a. and b. the main figure shows Plant (*P*_*C*_) biomass while the inset shows Herbivore (*H*_*C*_) biomass.

Here we explore the idea that sloppy grazing, a low *e1*, and the release of mineral nitrogen from the detrital pool can offset the negative effects of herbivory. However, to test this idea we must consider the outcome of this model in the absence of herbivores, as a control. As stated previously, stability analysis revealed three steady state solutions (S1 Text). In the second solution the herbivore is extinct. In this case, plant biomass increases through values of *r*_*P*_ (because plant N uptake is a function of *r*_*P*_) until the herbivore is present (Fig 4a). Once under the influence of herbivory, plant biomass does not increase any further. Once within the range of parameters in which herbivores exist, increasing herbivory, *r*_*1H*_, leads to declines in plant biomass, but the rate of decline rapidly slows (Fig 4b). The outcome of this analysis reveals the impact of herbivory, though moderated.

Our model was inspired by theoretical work carried out in soil food webs [33–36], and the parameter values were selected from our own greenhouse experiments and the soil food web literature. However, it is useful to consider the implications of this model for all plant herbivore interactions including grazing above-ground green biomass or the consumption of phytoplankton and vegetation in aquatic environments. The stoichiometric nature of this model allows for that possibility given the appropriate parameter values. Plants in aquatic environments tend to be of higher quality as they lack carbon rich support tissues (lignin) required for living on land [8]. This difference in aquatic and terrestrial plants may lead to a greater degree of nitrogen mineralization in aquatic systems than one would see for comparable plant biomass on land. Even though the priming influence of a pulse of organic matter is a general phenomenon that occurs in aquatic as well as terrestrial environments [37], the physical factors of living in water may interact with the fraction of plant biomass actually consumed. The spatial and temporal scale over which nutrient cycling is coupled to herbivory is likely shorter in a terrestrial than an aquatic environment [38]. Organic matter released through partial feeding under water may quickly diffuse away. Conversely in terrestrial soil, it is more likely to remain close to the rhizosphere. Further, consumption patterns may tip the balance to more nutrient cycling in the detrital pool for terrestrial plants and more herbivore driven nutrient cycling for aquatic plants. That is, in a pelagic aquatic environment, much of the primary producer biomass is in the form of phytoplankton, and it is usually consumed whole without the production of large amounts of detritus [39,40]. In our model, that would make *e*_*1*_ = 1.0. The flux of organic matter to the detrital pool would be limited to herbivore death and plant death (Fig 1). Our model does not account for differences in herbivore feeding strategies or optimal foraging. However, these would certainly interact with both *e*_*1*_ (*e*.*g*. a phloem sucking versus a chewing insect) and the stoichiometric quality of the food they select [29].

## Conclusions

The results of this model have implications for the way we think about plant herbivore interactions. Any time a herbivore is of higher quality (low C:N) consuming a plant of lower quality (high C:N) the herbivore is going to respire CO_2_, scavenge the nitrogen and drive plants into nitrogen limitation in that environment [41]. However, models show that the stoichiometric quality of the herbivore can pose restrictions depending on the plants own nutrient affinity (*sensu* Daufresne and Loreau [26]). As many plants are non-homeostatic with respect to C:N, this will eventually alter the quality of the plants’ own tissue. Our model does not account for non-homeostatic changes in long-term plant quality, but it does show that the fraction of plant biomass actually consumed and metabolized by the herbivore will affect the degree to which a plant can recoup any losses. Furthermore, neighboring plants benefit from local herbivory [42], and herbivory may have significant effects on competitive outcomes [43]. This model mechanistically connects the direct effects of herbivory with the indirect effects of soil nutrient cycling. It helps resolve the mechanisms associated with herbivory and feedbacks to primary production.

## Supporting Information

S1 Text Three Steady State Solutions. (PDF)

S2 Text Jacobial Matrix. (PDF)

S3 Text Model Criterion. (PDF)

## References

[1] Bardgett RD, Wardle DA (2010) Aboveground—Belowground Linkages: Biotic Interactions, Ecosystem Processes and Global Change: Oxford.

[2] Hamilton EW, Frank DA, Hinchey PM, Murray TR (2008) Defoliation induces root exudation and triggers positive rhizospheric feedbacks in a temperate grassland. Soil Biology & Biochemistry 40: 2865–2873.

[3] Ruess RW, McNaughton SJ (1987) Grazing and the dynamics of nutrient and energy regulated microbial processes in the Serengeti grasslands. Oikos 49: 101–110.

[4] Agrawal AA (2000) Overcompensation of plants in response to herbivory and the by-product benefits of mutualism. Trends in Plant Science 5: 309–313. 10871904 10.1016/s1360-1385(00)01679-4

[5] Bonkowski M (2004) Protozoa and plant growth: the microbial loop in soil revisited. New Phytologist 162: 617–631. 10.1111/j.1469-8137.2004.01066.x 33873756

[6] Cherif M, Loreau M (2013) Plant—herbivore—decomposer stoichiometric mismatches and nutrient cycling in ecosystems. Proceedings of the Royal Society B-Biological Sciences 280 doi: 10.1098/rspb.2013.1354 23303537 10.1098/rspb.2012.2453 PMC3574320

[7] Osler GHR, Sommerkorn M (2007) Toward a complete soil C and N cycle: Incorporating the soil fauna. Ecology 88: 1611–1621. 17645007 10.1890/06-1357.1

[8] Sterner RW, Elser JJ (2002) Ecological Stoichiometry. Princeton: Princeton University Press.

[9] Elser J, Fagan WF, Denno RF, Dobberfuhl DR, Folarin A, et al (2000) Nutritional constraints in terrestrial and freshwater food webs. Nature 408: 578–580. 11117743 10.1038/35046058

[10] Bardgett RD, Wardle DA (2003) Herbivore-mediated linkages between aboveground and belowground communities. Ecology 84: 2258–2268.

[11] Hamilton EW, Frank DA (2001) Can plants stimulate soil microbes and their own nutrient supply? Evidence from a grazing tolerant grass. Ecology 82: 2397–2402.

[12] Kuzyakov Y, Friedel JK, Stahr K (2000) Review of mechanisms and quantification of priming effects. Soil Biology & Biochemistry 32: 1485–1498.

[13] Mikola J, Setala H, Virkajarvi P, Saarijarvi K, Ilmarinen K, et al (2009) Defoliation and patchy nutrient return drive grazing effects on plant and soil properties in a dairy cow pasture. Ecological Monographs 79: 221–244.

[14] Anderson TR, Hessen DO, Elser JJ, Urabe J (2005) Metabolic stoichiometry and the fate of excess carbon and nutrients in consumers. The American naturalist 165: 1–15. 15729636 10.1086/426598

[15] Sih A (1980) Optimal foraging: partial consumption of prey. American Naturalist: 281–290.

[16] Lindeman RL (1942) The trophic-dynamic aspect of ecology. Ecology 23: 399–418.

[17] McNaughton SJ (1976) Serengeti migratory wildebeest: facilitation of energy flow by grazing. Science 191: 92–94. 17834943 10.1126/science.191.4222.92

[18] Bardgett RD, Denton CS, Cook R (1999) Below-ground herbivory promotes soil nutrient transfer and root growth in grassland. Ecology Letters 2: 357–360. 10427718

[19] Tu C, Koenning SR, Hu S (2003) Root-parasitic nematodes enhance soil microbial activities and nitrogen mineralization. Microbial Ecology 46: 134–144. 12739076 10.1007/s00248-002-1068-2

[20] van Veen JA, Ladd JN, Frissel MJ (1984) Modelling C and N turnover through microbial biomass in soil. Plant and Soil 76: 257–274.

[21] Vitousek PM, Howarth RW (1991) Nitrogen limitation on land and in the sea: how can it occur? Biogeochemistry 13: 87–115.

[22] Liebig J (1840) Chemistry and Its Application to Agriculture and Physiology. London: Taylor and Walton.

[23] Evans JR (1989) Photosynthesis and nitrogen relationships in leaves of C3 plants. Oecologia 78: 9–19. 28311896 10.1007/BF00377192

[24] Moore JC, McCann K, de Ruiter PC (2005) Modeling trophic pathways, nutrient cycling, and dynamic stability in soils. Pedobiologia 49: 499–510.

[25] Hoagland D, Arnon D (1938) The water culture method for growing plants with out soil. Berkley, CA: University of California Agriculture Experiment Station.

[26] Daufresne T, Loreau M (2001) Plant-herbivore interactions and ecological stoichiometry: when do herbivores determine plant nutrient limitation? Ecology Letters 4: 196–206.

[27] Van der putten WH, Vandijk C, Troelstra SR (1988) Biotic soil factors affecting the growth and development of *Ammophila arenaria* . Oecologia 76: 313–320. 28312214 10.1007/BF00379970

[28] Hillebrand H, Borer ET, Bracken MES, Cardinale BJ, Cebrian J, et al (2009) Herbivore metabolism and stoichiometry each constrain herbivory at different organizational scales across ecosystems. Ecology Letters 12: 516–527. doi: 10.1111/j.1461-0248.2009.01304.x 19392711 10.1111/j.1461-0248.2009.01304.x

[29] Cebrian J (1999) Patterns in the fate of production in plant communities. American Naturalist 154: 449–468. 10523491 10.1086/303244

[30] Knoll LB, McIntyre PB, Vanni MJ, Flecker AS (2009) Feedbacks of consumer nutrient recycling on producer biomass and stoichiometry: separating direct and indirect effects. Oikos 118: 1732–1742.

[31] Wardle DA, Bardgett RD, Klironomos JN, Setala H, van der Putten WH, et al (2004) Ecological linkages between aboveground and belowground biota. Science 304: 1629–1633. 15192218 10.1126/science.1094875

[32] Frost PC, Benstead JP, Cross WF, Hillebrand H, Larson JH, et al (2006) Threshold elemental ratios of carbon and phosphorus in aquatic consumers. Ecology Letters 9: 774–779. 16796566 10.1111/j.1461-0248.2006.00919.x

[33] de Ruiter PC, Neutel A-M, Moore JC (1995) Energetics, patterns adn interaction strengths and stability in real ecosystems. Science 269: 1257–1260. 17732112 10.1126/science.269.5228.1257

[34] Hunt HW, Coleman DC, Ingham ER, Ingham RE, Elliot ET, et al (1987) The detrital food web in a shortgrass prairie. Biology Fertility of Soil 3: 57–68.

[35] Moore JC, de Ruiter PC, Hunt HW, Coleman DC, Freckman DW (1996) Microcosms and soil ecology: critical linkages between field studies and modelling food webs. Ecology 77: 694–705.

[36] Raynaud X, Lata JC, Leadley PW (2006) Soil microbial loop and nutrient uptake by plants: a test using a coupled C: N model of plant-microbial interactions. Plant and Soil 287: 95–116.

[37] Guenet B, Danger M, Abbadie L, Lacroix G (2010) Priming effect: bridging the gap between terrestrial and aquatic ecology. Ecology 91: 2850–2861. 21058546 10.1890/09-1968.1

[38] Krumins JA, van Oevelen D, Bezemer TM, De Deyn GB, Hol WHG, et al (2013) Soil and Freshwater and Marine Sediment Food Webs: Their Structure and Function. Bioscience 63: 35–42.

[39] Chase JM (2000) Are there real differences among aquatic and terrestrial food webs? Trends in Ecology and Evolution 15: 408–412. 10998518 10.1016/s0169-5347(00)01942-x

[40] Cyr H, Pace ML (1993) Magnitude and patterns of herbivory in aquatic and terrestrial ecosystems. Nature 361: 148–150.

[41] Sterner RW (1990) The Ratio of nitrogen to phosphorus resupplied by herbivores—zooplankton and the algal competitive arena. American Naturalist 136: 209–229.

[42] Ayres E, Dromph KM, Cook R, Ostle N, Bardgett RD (2007) The influence of below-ground herbivory and defoliation of a legume on nitrogen transfer to neighbouring plants. Functional Ecology 21: 256–263.

[43] Daufresne T, Hedin LO (2005) Plant coexistence depends on ecosystem nutrient cycles: Extension of the resource-ratio theory. Proceedings of the National Academy of Sciences of the United States of America 102: 9212–9217. 15964989 10.1073/pnas.0406427102 PMC1166585

[44] Ahn M-Y, Zimmerman AR, Comerford NB, Sickman JO, Grunwald S (2009) Carbon mineralization and labile organic carbon pools in the sandy soils of a northern Florida watershed. Ecosystems 12: 672–685.

