# Supplementary material for: Herbivory and Stoichiometric Feedbacks to Primary Production
Source: PLoS One. 2015 Jun 22;10(6):e0129775. doi: 10.1371/journal.pone.0129775 (PMC4476572; doi:10.1371/journal.pone.0129775)
Supplement: S1 Text — (PDF) [file pone.0129775.s001.pdf]

S1 Supplement.

The steady state solution where all living pools are extinct:

$$H_C = 0, \qquad D_N = 0, \qquad D_C = 0, \qquad P_C = 0, \qquad N = \frac{N_{ext}}{K_{NL}}.$$

The steady state solution where the herbivore is extinct:

$$H_C = 0,\ D_N = \frac{N_{ext}}{K_{DL}} - \frac{K_{NL}d_P}{K_{DL}r_P},\ D_C = D_N CNP\left(1 + \frac{K_{DL}}{r_{min}}\right),\ P_C = \frac{D_C\, r_{min}}{d_P},\ \ N = \frac{d_P}{r_P}.$$

The steady state solution where all pools coexist:

$$P_C = \frac{d_H + r_{2H}}{e_1 r_{1H}}, \qquad N = \frac{r_{1H}H_C + d_P}{r_P},$$

$$H_C = \frac{e_1(r_{min} + k_{DL})(r_P N_{ext} - k_{NL}d_P) - \frac{1}{CNP}\frac{r_P}{r_{1H}} d_P k_{DL}(d_H + r_{2H})}{e_1 r_{1H} k_{NL}(r_{min} + k_{DL}) + r_P k_{DL}\left(\frac{(1-e_1)}{CNP}(d_H + r_{2H}) + \frac{e_1}{CNH} d_H\right)},$$

$$D_N = \frac{\frac{d_P(d_H + r_{2H})}{CNP \cdot e_1 \cdot r_{1H}} + \left(\frac{(1-e_1)(d_H + r_{2H})}{e_1 \cdot CNP} + \frac{d_H}{CNH}\right)H_C}{r_{min} + k_{DL}},$$

$$D_C = \left(\frac{d_H}{e_1 r_{min}} + \left(\frac{1}{e_1} - 1\right)\frac{r_{2H}}{r_{min}}\right)H_C + \frac{d_P(d_H + r_{2H})}{e_1 r_{1H} r_{min}}$$
