# Supplementary material for: Herbivory and Stoichiometric Feedbacks to Primary Production
Source: PLoS One. 2015 Jun 22;10(6):e0129775. doi: 10.1371/journal.pone.0129775 (PMC4476572; doi:10.1371/journal.pone.0129775)
Supplement: S2 Text — (PDF) [file pone.0129775.s002.pdf]

S2 Supplement.

$$\begin{bmatrix} r_P N - d_P - r_{1H} H_C & -r_{1H} P_C & r_P P_C & 0 & 0 \\ e_1 r_{1H} H_C & e_1 r_{1H} P_C - d_H - r_{2H} & 0 & 0 & 0 \\ e_1 r_{1H} \left(\frac{H_C}{CNP} - \frac{H_C}{CNH} \right) - \frac{r_P N}{CNP} & e_1 r_{1H} \left(\frac{P_C}{CNP} - \frac{P_C}{CNH} \right) + \frac{r_{2H}}{CNH} & -\frac{r_P P_C}{CNP} - K_{NL} & r_{\min} & 0 \\ \frac{d_P}{CNP} + \frac{(1-e_1) r_{1H} H_C}{CNP} & \frac{d_H}{CNH} + \frac{(1-e_1) r_{1H} P_C}{CNP} & 0 & -r_{\min} - K_{DL} & 0 \\ d_P + (1-e_1) r_{1H} H_C & d_H + (1-e_1) r_{1H} P_C & 0 & 0 & -r_{\min} \end{bmatrix}$$
