# Supplementary material for: Herbivory and Stoichiometric Feedbacks to Primary Production
Source: PLoS One. 2015 Jun 22;10(6):e0129775. doi: 10.1371/journal.pone.0129775 (PMC4476572; doi:10.1371/journal.pone.0129775)
Supplement: S3 Text — (PDF) [file pone.0129775.s003.pdf]

S3 Supplement.

Excretion, as defined in Figure 1, must be greater than or equal to zero, as herbivores cannot take up inorganic N from the environment. Because herbivore excretion must be non-negative, the relative C:N ratios of plants and herbivores and herbivore metabolism are constrained as follows:

$$\frac{CNH}{CNP} + \frac{r_{2H}}{e_1 r_{1H} P_C} \geq 1$$

This criterion is intuitive biologically. As the ratio of *CNH/CNP* decreases, the herbivore must respire the excess carbon as CO_2. Therefore the model is sensitive to the values of r_{2H}, *CNP* and *CNH*. High values of herbivore C:N or of herbivore respiration relative to plant matter ingestion, or both, contribute to this criterion being satisfied.
